# The Efficiency of CBD Production Using Grafted *Cannabis sativa* L. Plants Is Highly Dependent on the Type of Rootstock: A Study

**DOI:** 10.3390/plants13081117

**Published:** 2024-04-17

**Authors:** Luka Bitežnik, Roman Štukelj, Marko Flajšman

**Affiliations:** 1 Department of Agronomy, Biotechnical Faculty, University of Ljubljana, Jamnikarjeva 101, SI-1000 Ljubljana, Slovenia; luka.biteznik@bf.uni-lj.si; 2 Research Institute, Faculty of Health Sciences, University of Ljubljana, Zdravstvena pot 5, SI-1000 Ljubljana, Slovenia; roman.stukelj@zf.uni-lj.si

**Keywords:** *Cannabis sativa* L., cannabinoids, grafting, rootstock, scion

## Abstract

The global cannabis market is continuously expanding and as a result, the cannabis industry demands new and improved agronomic cultivation practices to increase production efficiency of cannabidiol (CBD), which is valued for its therapeutic benefits. This study investigates the influence of three rootstock types on the survival rate, morphological parameters, and biochemical composition of cannabis: potentially dwarfing rootstocks (PDR), potentially vigorous rootstocks (PVR), and seedlings-as-rootstocks (SAR). Rootstocks were used for grafting two scion genotypes: ‘ScionII’ = chemotype II of industrial hemp, and ‘ScionIII’ = chemotype III of high CBD accumulating variety. Contrary to expectations, PVR and SAR did not outperform PDR on most of the measured variables. SAR showed the highest survival rate of the grafted cannabis plants (40–70%). The rootstock type had a statistically significant influence only on the bud compactness index in ‘ScionII’, with PDR being particularly noticeable. A comparative analysis of the ‘rootstock/scion’ combinations with their controls (non-grafted scions) revealed grafting’s substantial improvement in most traits. Specifically, PDR increased CBD content by 27% in ‘ScionIII’, inflorescence yield and CBD yield per plant increased by 71% and 84%, respectively, when SAR was used in ‘ScionII’. SAR showed to be the most effective rootstock type for CBD production. Our findings suggest grafting as a promising technique for optimizing cannabis’s agronomic and medicinal potential, highlighting the necessity for further research on its underlying mechanisms to refine production efficiency and quality.

## 1. Introduction

Medical cannabis and hemp (*Cannabis sativa* L.) contain many bioactive compounds with biological effects in the human body, and as a result, it is becoming important in the pharmaceutical industry and medicine [1]. Cannabinoids or phytocannabinoids represent largest group of secondary metabolites in cannabis plant. The dominant ones are tetrahydrocannabinolic acid (THCA), cannabidiolic acid (CBDA), cannabigerolic acid (CBGA), cannabinolic acid (CBNA), and cannabichromenic acid (CBCA) [2,3]. Cannabis is divided into four chemotypes based on the ratio of the neutral cannabinoids: chemotype I contains high concentrations of the cannabinoid tetrahydrocannabinol (THC) (>0.3%) and low concentrations of cannabidiol (CBD) (<0.5%) with THC/CBD > 1; chemotype II contains a lot of THC (>0.3%) and high CBD (>0.5%) with THC/CBD ≈ 1; chemotype III contains high CBD (>0.5%) and low THC (<0.3%) with THC/CBD < 1 [4,5,6,7]. Subsequently, in 1987, Fournier et al. identified chemotype IV, which contains over 0.3% cannabigerol (CBG) [8].

The goals of high-quality cannabis cultivation include a consistent and uniform yield of inflorescences and the production of these specific secondary metabolites. Therefore, professional medical cannabis producers are transitioning from greenhouses into more controlled and closed indoor growth chambers [9]. Indoor cannabis cultivation is one of the most energy-intensive industries to maintain [10], with the main energy consumption requirements of indoor production facilities being the intense lighting, dehumidification, space heating or cooling, drying, pre-heating of irrigation of water, generation of carbon dioxide, and 30 hourly air changes. This consumption results in power densities of 2000 W/m^2^, which is comparable to modern computer data centers [11].

The bioactive substances of cannabis are mainly produced by unfertilized female plants, meaning only female plants are commercially cultivated for the production of secondary metabolites. Female plants can be obtained using feminized seeds [12], but due to heterogeneity of seedlings, selected female plants are usually vegetatively propagated to achieve uniform yields and easier production [2]. The rooting success rate in vegetative propagation by stem cuttings depends on many factors such as humidity [13], temperature [14], length and thickness of shoots [15], age and condition of mother plant [16], and genotype. The formation of adventitious roots is genotype-specific and determines the ability for successful vegetative propagation [17].

Grafting is an ancient, vegetative propagation technique. It involves the conjunction of at least two plant parts—root system (rootstock) and shoot (scion)—that form a grafted plant. Rootstocks can alter the biochemical and physiological processes of the scion and change plant tolerance to biotic and abiotic stresses [18]. It may serve as a source of endogenous plant hormones, thus leading to increased growth and yield of the scion in addition to disease control [19]. It has been shown that grafting exhibits excellent tolerance to serious soil-borne diseases. For instance, grafted watermelons show increased resistance to *Fusarium* L. and *Verticillium* N., bell peppers to *Phytophthora* B., and melons to *Didymella bryoniae* R., showing grafting’s role in tolerating diseases [20,21].

Choosing the right rootstock for a particular scion is crucial for the success of the grafted plants, as the rootstock has a direct influence on the survival rate, growth parameters, and the quality of the harvest. Rootstocks impact various growth parameters, including plant biomass, leaf area, and the length of generative development in different crops [22,23,24]. This underscores the critical importance of meticulous rootstock selection in grafting practices.

Plants grown from seeds usually have a single primary root (the taproot), while plants grown from cuttings have several primary roots. The total root mass of seedlings is higher than that of cuttings, resulting in differences in root mass and soil exploration ability [25,26]. Rootstocks can be dwarfing, in which they reduce vigour of the scion, or vigorous, which increases the vigour of the scion [27,28]. When grafting tomato (*Solanum lycopersicum* L.), eggplant (*Solanum melongena* L.), and bell pepper (*Capsicum annuum* L.) plants, seedlings are used as rootstocks and scions, and it is important that they are healthy and uniform in size at the time of grafting [29,30,31]. Studies have shown that the method of propagation influences root architecture, with stem cuttings developing shallower roots compared to seedlings [32,33,34].

To this date, the only known rule for grafting cannabis is that both the rootstock and the scion must be clonally propagated from vegetative tissue [35]. This method is unique compared to other non-woody plants, where seedlings are usually used as rootstocks. Grafting with clonally propagated germplasm differs from conventional vegetative propagation with cuttings and has not yet been studied in detail. Unpublished experiments at our institution have shown that certain genotypes cannot be vegetatively propagated by stem cuttings easily. Grafting offers an alternative for the propagation of these problematic genotypes. However, as research has shown [36], the survival rates of grafted plants can also differ significantly between varieties and grafting can affect plant survival. One reason for researching cannabis grafting is the interest in gaining knowledge about this technique and optimising the vegetative propagation of cannabis.

Despite the growing interest in cannabis cultivation techniques, research on the application of grafting in cannabis production remains scarce, particularly in terms of effects on plant morphology, yield, and secondary metabolite concentrations. This gap underscores the urgent need for comprehensive studies investigating the efficacy of grafting in cannabis. Research on various plant species confirms that grafting can modify the secondary metabolism, leading to enhanced production of bioactive compounds [37,38,39,40]. The special physiological and biochemical requirements of cannabis call for targeted research. This should investigate how different combinations of rootstock and scion affect the production of important medicinal compounds, including CBD. Our study attempts to fill this knowledge gap and provide insights that could lead to significant advances in the field of medical cannabis.

To the best of our knowledge, the method for grafting cannabis using different rootstock types, including seedlings as rootstock, has not yet been described. By pioneering research into the use of seedlings as rootstock for cannabis grafting, this study lays the foundation for the development of cultivation strategies that could improve the sustainability and efficiency of cannabis production. The aims of our study were to (i) compare the effectiveness/success rate of obtaining grafted plants using different rootstock types (categorised as potentially vigorous, potentially dwarfing, and derived from seedlings) and (ii) evaluate the influence of these different rootstock types on the morphological and biochemical performance of grafted cannabis plants using two different scion genotypes in indoor experiment.

## 2. Results

### 2.1. Success of Two-Step Grafted Plant Preparation

Table 1 shows the rooting success rate and subsequent survival of the scion genotype ‘ScionII’ when grafted onto different rootstocks. This includes three different phases: number of rootstocks (NR), number of successfully rooted rootstocks (RR), and number of surviving plants post-grafting (SP). Each of the clonally propagated rootstocks started with 13 rootstocks. In the potentially vigorous rootstock group (PVR), the combination ‘Vigor-C23/ScionII’ had the lowest RR rate of about 54% (7 out of 13), with a subsequent survival rate of 71% (5 out of 7) of the grafted plants (SP). In contrast, ‘Vigor-S39/ScionII’ had a higher RR of about 92% (12 out of 13), but a lower SP of 25% (3 out of 12). In the potentially dwarfing rootstock (PDR) group, each combination had a similar RR but different SP from 2 to 4 plants. The seedling-as-rootstock combinations (SAR) showcased a perfect RR, with the number of SP ranging from 4 to 7. Finally, the control group (‘Control/ScionII’) had a SP of 63% (5 out of 8) after acclimatisation.

Regarding the scion genotype ‘ScionIII’ (Table 2), the combination ‘Vigor-S26/ScionIII’ clearly outperformed the others with the highest RR rate. However, combination (‘Vigor-MER/ScionIII’) had the lowest SP of the group. Within the PDR group, ‘Dwarf-T34/ScionIII’ had the highest RR at 83% (10 out of 12). The SP was consistent with the other combinations within this group. The SAR combinations achieved flawless rootstock obtainment, while the SP was between 4–5 plants. Finally, the control group (‘Control/ScionIII’) had a low RR but showed a perfect SP after the acclimatization process.

### 2.2. Influence of Rootstock on Morphological, Biochemical and Yield Parameters of Cannabis Plants

The statistical analysis revealed only one statistically significant influence of the rootstock type on the selected morphological variable. Otherwise, no other statistically significant influence was found. However, relative comparison of the results gave interesting outcome, therefore we present the results as the unweighted index number (UIN), which explain the percentage change in the values of different variables [41]. Each combination ’Rootstock/Scion’ was compared to control combination (non-grafted scion genotype) of the same scion genotype. Detailed results of real measurements are available in Supplementary Table S1.

#### 2.2.1. Influence of Rootstock on Morphological Variables of Grafted Cannabis Plants

**Figure 1:**
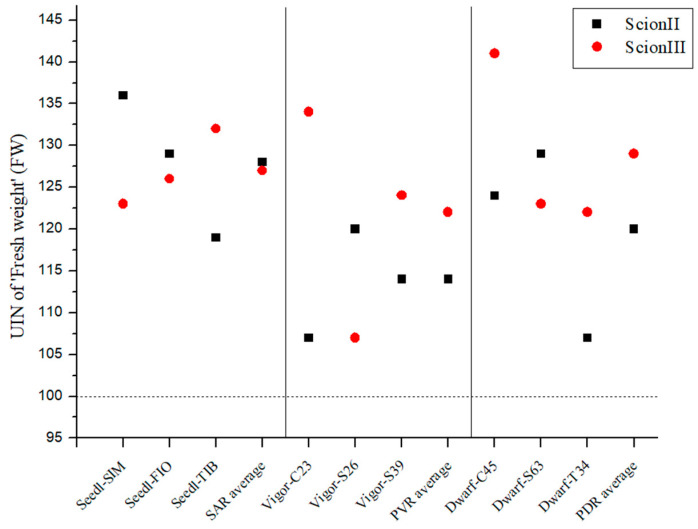
Fresh weight (FW) of two plant scion genotypes grafted on 9 different rootstocks expressed as UIN, where value of 100 represent control combination (non-grafted plants). SIM, S—population ‘Simba’, FIO—variety ‘Fiona’, TIB, T—variety ‘Tiborszallasi’, C—variety ‘Carmagnola’, Seedl, SAR—seedling-as-rootstock combination, Vigor, PVR—potentially vigorous rootstocks, Dwarf, PDR—potentially dwarfing rootstocks, ScionII—scion of chemotype II, and ScionIII—scion of chemotype III.

Figure 1 shows a remarkable increase in biomass growth in all grafted plants compared to the control group (non-grafted plants). When looking at the ‘ScionII’ scion genotype, the seedling-as-rootstock combinations (SAR) exhibited the most substantial effect on growth, with an average UIN of 128, corresponding to a 28% increase in fresh weight (FW). It is noteworthy that the ‘Seedl-SIM/ScionII’ combination achieved the highest UIN (139) compared to the control group (‘Control/ScionII’). The potentially vigorous rootstocks (PVR) group had the lowest average UIN. The data indicate that potentially dwarfing rootstocks (PDR) have a stronger influence on the FW of the plants than PVR. For the scion genotype ‘ScionIII’, our results show that PDR enabled the largest average increase in fresh weight compared to the other rootstock types. It is noteworthy that the combination ‘Dwarf-C45/ScionIII’ had the highest UIN value of 141, which corresponded to a 41% increase in FW. In contrast, PVR had the lowest average UIN, while the ‘Vigor-C45/ScionIII’ combination had the greatest impact on FW. The SAR combinations also showed an increased FW of the plants compared to the control group (‘Control/ScionIII’).

The genotype of the rootstock had a significant influence on the bud compact index (BCI) of the scion genotype ‘ScionII’ (*p* = 0.0158). All three genotypes of the PDR type had the highest BCI values (0.30–0.24 g/cm) and the difference was statistically significant compared to ‘Control/ScionII’ (0.12 g/cm). In terms of PVR, only the ‘Vigor-S26/ScionII’ combination (0.22 g/cm) showed a statistically significant difference in BCI compared to the control group. Within the SAR group, the combination ‘Seedl-SIM/ScionII’ (0.21 g/cm) showed the highest increase in BCI compared to the control group. None of the combinations within this rootstock group showed a significant difference compared to the control group (Table 3).

**Figure 2:**
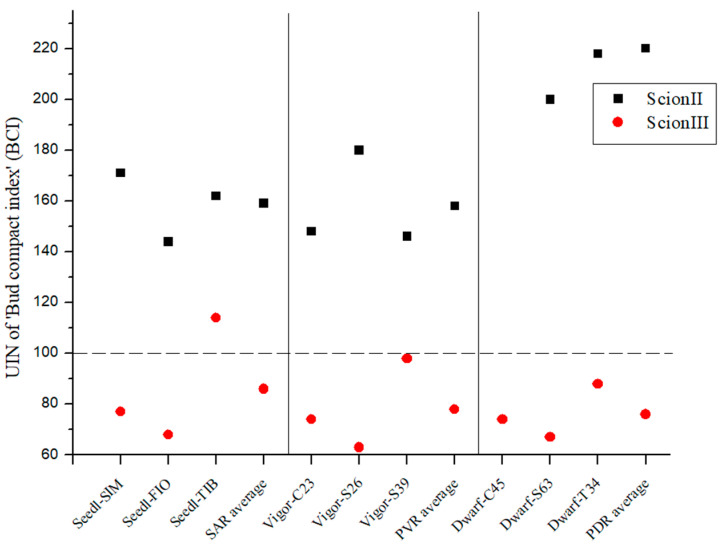
Bud compact index of two plant scion genotypes grafted on 9 different rootstocks expressed as UIN, where value of 100 represent control combination (non-grafted plants). SIM, S—population ‘Simba’, FIO—variety ‘Fiona’, TIB, T—variety ‘Tiborszallasi’, C—variety ‘Carmagnola’, Seedl, SAR—seedling-as-rootstock combination, Vigor, PVR—potentially vigorous rootstocks, Dwarf, PDR—potentially dwarfing rootstocks, ScionII—scion of chemotype II, and ScionIII—scion of chemotype III.

Regarding UIN for ‘ScionII’ genotype, PDR caused a 120% for BCI, where SAR indicated 59% and PVR 58% increase (Figure 2).

In the case of the genotype ‘ScionIII’, no statistically significant influence of the genotype of the rootstock on the BCI was observed. The majority of combinations exhibited UIN values below 100, which means that BCI values were lower compared to the control (non-grafted plants). Only the combination ‘Seedl-TIB/ScionIII’ showed an increase in UIN compared to the control group (Figure 2).

#### 2.2.2. Influence of Rootstock on Biochemical Variables of Grafted Cannabis Plants

**Figure 3:**
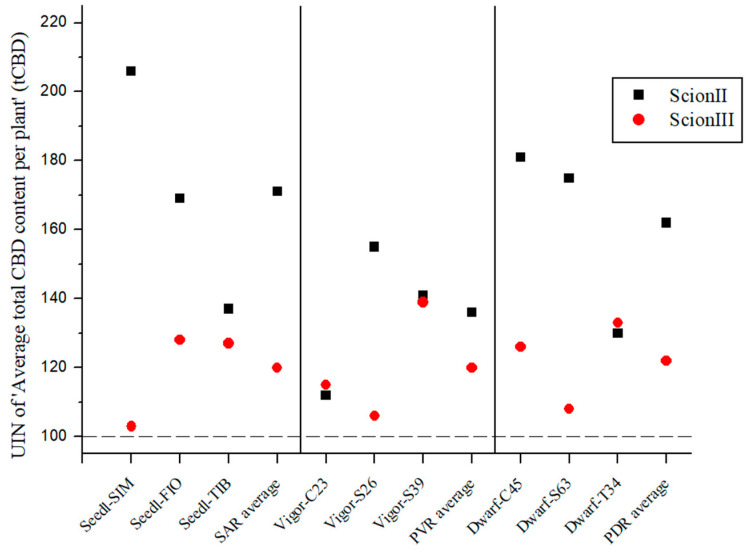
Average total CBD content per plant (tCBD) for two scion genotypes grafted on nine different rootstocks expressed as UIN, where value of 100 represent control combination (non-grafted plants). SIM, S—population ‘Simba’, FIO—variety ‘Fiona’, TIB, T—variety ‘Tiborszallasi’, C—variety ‘Carmagnola’, Seedl, SAR—seedling-as-rootstock combination, Vigor, PVR—potentially vigorous rootstocks, Dwarf, PDR—potentially dwarfing rootstocks, ScionII—scion of chemotype II, and ScionIII—scion of chemotype III.

Figure 3 demonstrates that the total cannabidiol (tCBD) content was influenced by the rootstock and was dependent on both the rootstock and the scion. Although no statistically significant differences were found between combinations, the UIN suggests that rootstocks influence tCBD content in female inflorescences. In the ‘ScionII’ grafting, the use of SAR yielded the highest average UIN of 114, representing a 14% increase in tCBD content compared to the control group. Of these combinations, the ‘Seedl-TIB/ScionII’ combination showed the highest increase in tCBD content. Surprisingly, the PDR had a minimal effect on the average tCBD content, including the ‘Dwarf-S63/ScionII’ combination, which showed a decrease in tCBD content. The ‘Dwarf-C45/ScionII’ combination had the highest UIN value and contributed the most to tCBD with a 15% increase in tCBD content. In the PVR group, all combinations also showed a small increase in tCBD. With respect to the scion genotype ‘ScionIII’, data indicate that PDR produced the highest average UIN of 127 compared to SAR and PVR, resulting in an average increase in plant tCBD of 27% compared to the control group. Notably, the ‘Dwarf-C45/ScionIII’ combination represented the largest increase in tCBD with an increase of 45%, marking the most substantial tCBD increase observed in this study. PVR had almost no effect on tCBD levels. Interestingly, the ‘Vigor-MER/ScionIII’ combination showed an unexpected decrease in tCBD levels, while the SAR group showed comparable results to the ‘ScionII’ scion genotype.

**Figure 4:**
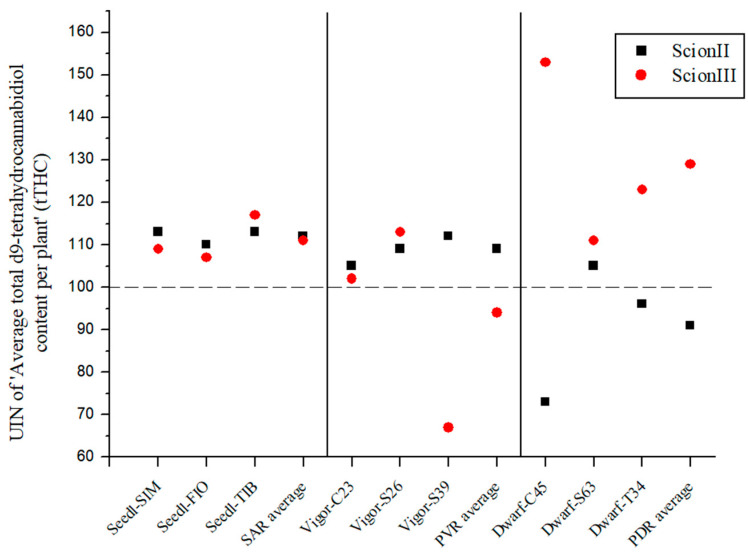
Average total d9-tetrahydrocannabidiol content per plant (tTHC) for two scion genotypes grafted on nine different rootstocks expressed as UIN, where value of 100 represent control combination (non-grafted plants). SIM, S—population ‘Simba’, FIO—variety ‘Fiona’, TIB, T—variety ‘Tiborszallasi’, C—variety ‘Carmagnola’, Seedl, SAR—seedling-as-rootstock combination, Vigor, PVR—potentially vigorous rootstocks, Dwarf, PDR—potentially dwarfing rootstocks, ScionII—scion of chemotype II, and ScionIII—scion of chemotype III.

The results shown in Figure 4 also indicate that rootstock can strongly influence the UIN of total THC content (tTHC) in ‘ScionII’ and ‘ScionIII’ plants. For the ‘ScionII’ genotype, the results indicate a significant influence of rootstock on tTHC.

The SAR combinations (Figure 4) resulted in an increase in average UIN with the highest individual increase of 13% (combination ‘Seedl-SIM/ScionII’). The PVR combinations only showed an increase in tTHC content in the ‘ScionII’ genotype. However, the PDR combinations led to a decrease in tTHC content for mentioned scion genotype. The combination ‘Dwarf-C45/ScionII’ led to the highest decrease of 27%. In the ‘ScionIII’ genotype, the PDR combinations had the highest average UIN value. The ‘Dwarf-C45/ScionIII’ combination had the highest UIN value of 153, which represented a 53% increase in tTHC content. These results show the decisive influence of rootstock on tTHC content in the ‘ScionIII’ genotype. The PVR combinations indicate a decrease in tTHC content, but the combinations had different results.

In this study we were able to detect 12 different terpenes, however their overall levels were low. ‘ScionIII’ showed higher terpene levels compared to ‘ScionII’, with the SAR type causing 0.48 wt% β-cis Ocimene and 0.47 wt% β-Caryophyllene. Only β-Caryophyllene and α-Humulene were increased in ‘ScionIII’ in all grafts compared to the control. Otherwise, no clear trend on the influence of grafting on terpene content was observed in this scion genotype. The terpene content of the ‘ScionII’ genotype was much lower compared to the ‘ScionIII’ genotype, with β-cis Ocimene (0.17 wt%) and α-Pinene (0.036 wt%) being the highest in the SAR type. Interestingly, all three rootstock types showed increased terpene content compared to the control combination ‘Control/ScionII’ (Supplementary Tables S2 and S3).

#### 2.2.3. Influence of Rootstock on Yield Parameters of Grafted Cannabis Plants

**Figure 5:**
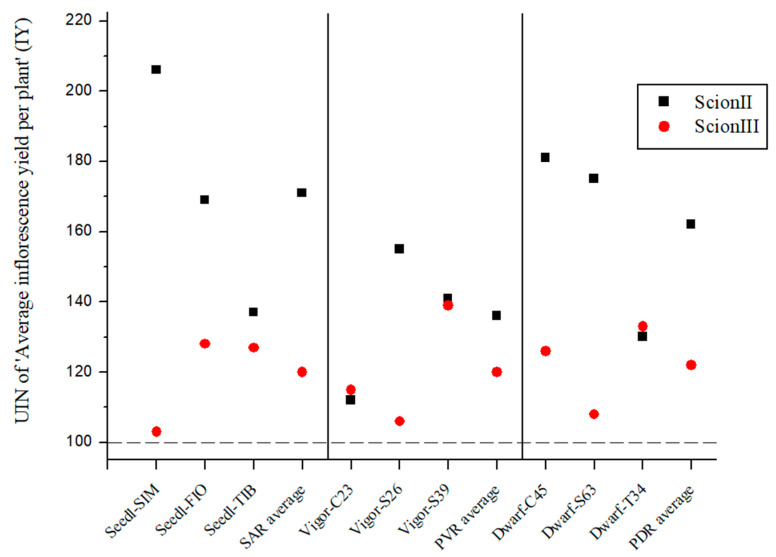
Average inflorescence yield per plant (IY) for two scion genotypes grafted on nine different rootstocks expressed as UIN, where value of 100 represent control combination (non-grafted plants). SIM, S—population ‘Simba’, FIO—variety ‘Fiona’, TIB, T—variety ‘Tiborszallasi’, C—variety ‘Carmagnola’, Seedl, SAR—seedling-as-rootstock combination, Vigor, PVR—potentially vigorous rootstocks, Dwarf, PDR—potentially dwarfing rootstocks, ScionII—scion of chemotype II, and ScionIII—scion of chemotype III.

Figure 5 presents the average UIN for inflorescence yield (IY) per combination. Although the differences between combinations were not statistically significant, the rootstocks selected appear to have influenced female inflorescence yield. For ‘ScionII’ grafted onto seedlings, the average UIN was 171, representing a 71% increase in IY. The ‘Seedl-SIM/ScionII’ combination had the highest UIN at 206, which represents a 106% increase in IY. PVR group had the least effect on IY, while in PDR group, the ‘Dwarf-C45/ScionII’ combination with a UIN of 181, showed an 81% increase in IY compared to the control. For ‘ScionIII’, rootstock genotype had a less pronounced effect on IY. ‘Dwarf-T34/ScionIII’ from the PDR combinations and ‘Vigor-MER/ScionIII’ from the PVR combinations showing notable increases in IY, the latter showing a 39% increase over the control. Overall, the data suggest that rootstock genotype influences inflorescence yield, with different combinations showing varying degrees of increase in IY compared to the control group.

**Figure 6:**
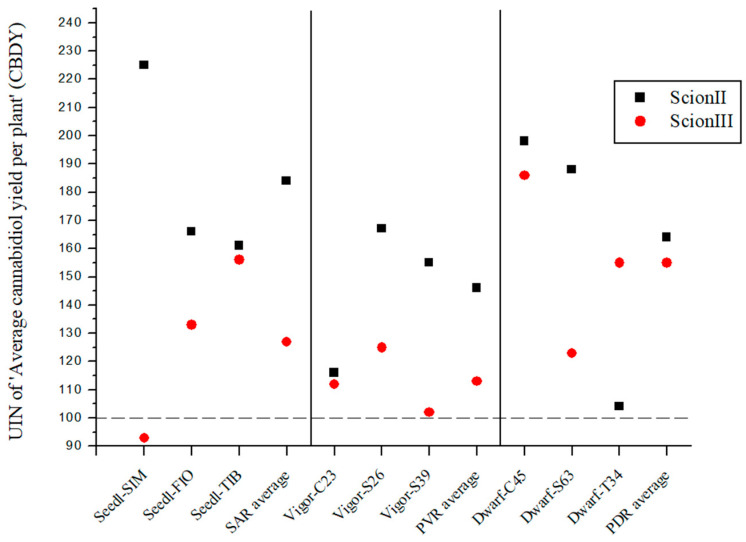
Average cannabidiol yield per plant (CBDY) for two plant scion genotypes grafted on nine different rootstocks expressed as UIN, where value of 100 represent control combination (non-grafted plants). SIM, S—population ‘Simba’, FIO—variety ‘Fiona’, TIB, T—variety ‘Tiborszallasi’, C—variety ‘Carmagnola’, Seedl, SAR—seedling-as-rootstock combination, Vigor, PVR—potentially vigorous rootstocks, Dwarf, PDR—potentially dwarfing rootstocks, ScionII—scion of chemotype II, and ScionIII—scion of chemotype III.

For the genotype ‘ScionII’, the results indicate that the rootstock significantly affects the CBD yield (CBDY) of the cannabis plants (Figure 6). The SAR combinations had an average UIN value of 184, which corresponds to an 84% increase in CBDY. The ‘Seedl-SIM/ScionII’ combination gave the highest UIN of 225, an increase of 125%. In the PVR combination group, the variety ‘Vigor-S26/ScionII’ showed the highest UIN value for CBDY. Within the PDR combinations the strongest effect on CBDY was observed in the ‘Dwarf-C45/ScionII’ and ‘Dwarf-S63/ScionII’ combinations. In comparison, the PDR combinations using ‘ScionIII’ as scion resulted in a considerable average UIN of 155, representing a 55% increase in CBDY. In particular, the ‘Dwarf-C45/ScionIII’ combination showed the highest increase of 86%. CBDY was also affected in the SAR and PVR groups.

### 2.3. Efficiency of CBD Production (PE_CBD_) Using Grafted Plants Is Highly Dependent on Rootstock Selection

**Figure 7:**
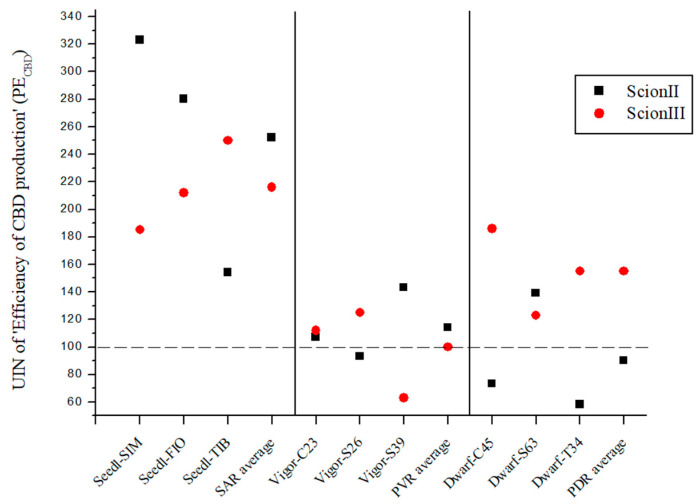
Efficiency of CBD production (PE_CBD_) for two plant scion genotypes grafted on nine different rootstocks expressed as UIN, where value of 100 represent control combination (non-grafted plants). SIM, S—population ‘Simba’, FIO—variety ‘Fiona’, TIB, T—variety ‘Tiborszallasi’, C—variety ‘Carmagnola’, Seedl, SAR—seedling-as-rootstock combination, Vigor, PVR—potentially vigorous rootstocks, Dwarf, PDR—potentially dwarfing rootstocks, ScionII—scion of chemotype II, and ScionIII—scion of chemotype III.

PE_CBD_ was created to evaluate the efficiency of CBD production using different rootstock types. The PE_CBD_ was calculated by dividing the total CBD yield of a given rootstock genotype by the number of rootstocks prepared for each treatment. It was than compared to the control, where the total cannabidiol yield of non-grafted grafted plants was divided by the number of rootstocks prepared for the control treatment. The data presented in Figure 7 show the substantial influence of rootstock selection on the production efficiency of cannabidiol (PE_CBD_), using UIN and expressed as a percentage increase compared to the control group.

In the genotype ‘ScionII’ grafted on SAR (seedling-as-rootstock), the average UIN was 252, representing an increase of 152% in PE_CBD_. In particular, the combination ‘Seedl-SIM/ScionII’ gave the highest UIN of 323. The ‘Seedl-FIO/ScionII’ combination recorded a UIN of 280, while the ‘Seedl-TIB/ScionII’ combination had the lowest UIN increase in PE_CBD_. In the PVR (potentially vigorous rootstocks), the average UIN value was much lower compared to the SAR group, indicating a small increase in PE_CBD_. The PDR combinations (potentially dwarfing rootstocks) gave the lowest average UIN value, indicating a decrease in PE_CBD_ for the ‘ScionII’ genotype. For the ‘ScionIII’ genotype, the SAR combinations had an average UIN value of 216, representing an increase in PE_CBD_ of 116%. Among these combinations, the ‘Seedl-TIB/ScionIII’ combination had the highest UIN of 250. Among the PDR combinations, the ‘Dwarf-C45/ScionII’ combination had the highest UIN value. Finally, the PVR combinations had an average UIN value indicating no difference in PE_CBD_ compared to the control group. The results emphasise the remarkable effect of rootstock selection on the efficiency of cannabidiol production in grafted cannabis plants.

### 2.4. Comparison of the Influence of Rootstock Type on Measured Variables by Scion Genotype

#### 2.4.1. ‘ScionII’ Genotype

**Figure 8:**
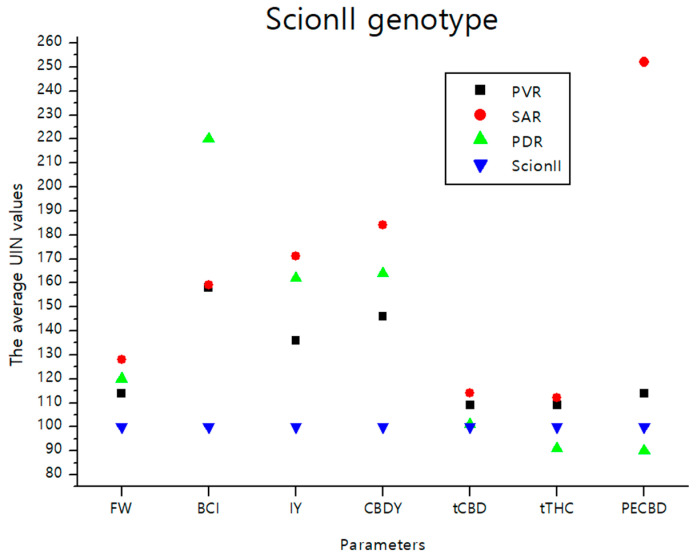
The average UIN values of parameters including FW, BCI, IY, CBDY, tCBD, tTHC, and PE_CBD_, of PVR, SAR, and PDR groups in comparison to the ‘Control/ScionII’ group, where value of 100 represent control combination (non-grafted plants). PVR—potentially vigorous rootstocks, SAR—seedling-as-rootstock combinations, PDR—potentially dwarfing rootstocks, ScionII—scion of chemotype II, FW—fresh weight, BCI—bud compact index, IY—average inflorescence yield per plant, CBDY—average CBD yield per plant, tCBD—average total CBD content per plant, tTHC—average total THC content per plant, PE_CBD_—efficiency of CBD production.

The data illustrated in Figure 8 consolidate the findings detailed in the previous sections. It visually depicts the effects of various rootstocks on the average UNI values of parameters including FW (fresh weight), BCI (bud compact index), IY (inflorescence yield), CBDY (CBD yield), tCBD (total CBD content), tTHC (total THC content), and PE_CBD_ (efficiency of CBD production) in comparison to the ‘Control/ScionII’ group. Notably, the rootstock PDR (potentially dwarfing rootstocks) demonstrated the most pronounced influence on BCI. Meanwhile, the parameter PE_CBD_ showed substantial responsiveness when a seedling was used as a rootstock for the ‘ScionII’ scion genotype.

#### 2.4.2. ‘ScionIII’ Genotype

**Figure 9:**
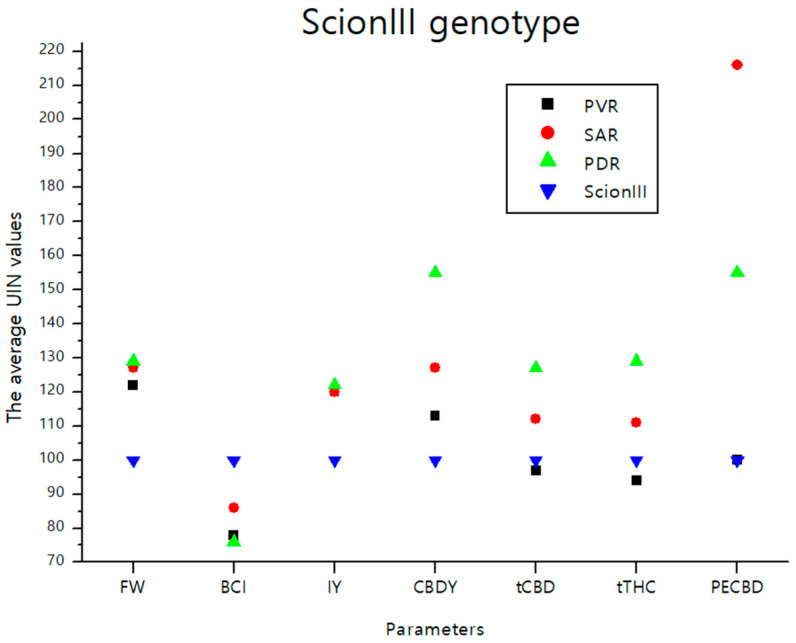
The average UIN values of parameters including FW, BCI, IY, CBDY, tCBD, tTHC, and PE_CBD_, of PVR, SAR and PDR groups in comparison to the ‘Control/ScionIII’ group, where value of 100 represent control combination (non-grafted plants). PVR—potentially vigorous rootstocks, SAR—seedling-as-rootstock combinations, PDR—potentially dwarfing rootstocks, ScionIII—scion of chemotype III, FW—fresh weight, BCI—bud compact index, IY—average inflorescence yield per plant, CBDY—average CBD yield per plant, tCBD—average total CBD content per plant, tTHC—average total THC content per plant, PE_CBD_—efficiency of CBD production.

The data presented in Figure 9 summarizes the findings from the previous section. It provides a graphical representation of the effects of different rootstocks on key growth and chemical parameters of cannabis plants from the ‘ScionIII’ scion. The graph illustrates the effects of the different groups of rootstocks (PVR, SAR, PDR) on the average UNI values of parameters FW, BCI, IY, CBDY, tCBD, tTHC, and PE_CBD_ compared to the ‘Control/ScionIII’ group. A notable observation is that the rootstock PDR exerted the greatest influence on CBDY. Conversely, the PE_CBD_ parameter showed a marked influence when a seedling was used as rootstock for the scion genotype ‘ScionIII’.

## 3. Discussion

The percentage of successfully rooted cuttings depends on various factors. The genetic basis and the age of the plant material have the greatest influence on the formation of adventitious roots [42,43]. Additional factors include leaf area of the cuttings, the position of the cutting on the mother plant, the concentration of endogenous hormones, the light intensity, the rooting medium, the water content, and the nutrient consumption [44,45]. Regarding survival rates in our grafting experiment, the data showed variability depending on the rootstock used. For the potentially dwarfing rootstocks (PDR) and potentially vigorous rootstocks (PVR) obtained as clones from selected mother plants, it is interesting to note that in some cases a high success rate in rooting the rootstocks did not necessarily lead to a high survival rate after grafting. For example, in ‘Vigor-S26/ScionII’, 12 out of 13 rootstock plants were rooted (Table 1) but only 3 grafted plants survived. In addition, ‘Vigour-S26/ScionIII’ had 11 out of 12 rooted plants and only 3 grafted plants survive (Table 2). This indicates that successful rooting of rootstocks is not the only factor determining graft survival. In some combinations, rooting success was low, with only 3 out of 14 in the ‘Control/ScionIII’ combination. Apparently, the ‘ScionIII’ genotype itself has poor rooting ability and by using the grafting technique it is also possible to improve root development in genotypes with poor rooting ability [35]. Further research could focus on understanding the reasons for these discrepancies in survival rates and possibly improving grafting techniques to increase survival rates.

In contrast, seedling-as-rootstocks (SAR) means no loss of plant material during rootstock establishment and a higher efficiency in obtaining grafted plants compared to other combinations as well as to self-rooted (non-grafted) controls. At the same time, it must be considered that the use of SAR does not require special cultivation techniques. Mother plants require a consistent and controlled environment with optimal light, temperature, and humidity conditions to ensure healthy growth and propagation by stem cuttings [35], which is less stringent when using SAR. However, when seedlings are used for rootstocks, high germination rate of rootstock seeds, homogeneous emergence of rootstock seeds, and rootstock/scion compatibility are important factors [46,47].

Taking all combinations together, the average survival rate of grafted plants was 38.1% for the scion ‘ScionIII’ and 29.5% for ‘ScionII’. In contrast to Purdy et al. (2022) [35], who used a one-step grafting method and reported a survival rate of up to 100%, a two-step grafting method was used in our experiment. They found that the older the rootstock, the lower the survival rate. In other words, the survival rate was highest when a fresh scion was grafted onto a fresh rootstock. It is possible that the two-step grafting method is less appropriate for cannabis grafting and one-step grafting is more suitable for obtaining a larger number of viable plants, which was also observed in our further (unpublished) experiments. However, SAR can only be used in the two-step grafting method.

The analysis of the biomass distribution revealed considerable differences depending on the combination of rootstock and scion. We expected that the different rootstock types would have the most pronounced and visible effects on the morphological characteristics of the grafted cannabis plants. Numerous studies have shown that certain rootstocks can increase both plant biomass weight and its fruit yields [48,49,50,51]. It is suggested that a more developed root system of vigorous rootstocks and root architecture can influence various physiological functions. These include improving water uptake, nutrient uptake, and subsequent transport to the scion, possibly leading to increased plant growth [48,52,53,54]. Turhan et al. (2011) [55] demonstrated that grafting tomatoes on suitable rootstocks significantly improves fruit yield, size, and number of fruits per truss compared to non-grafted plants. However, no significant differences were found between the different grafting combinations in terms of fruit characteristics. The improvement in growth and yield depends on the characteristics of the rootstock and its ability to modify the physiology of the scion [56,57]. However, as shown by Doñas-Uclés et al. (2015) [58], there is no clear correlation between yield and vigor conferred to the bell pepper (*Capsicum annum* L.) plant by the rootstock. In this context, it is crucial to understand the influence of rootstock on shoot growth, as rootstock can significantly influence hormone levels, which are closely linked to plant productivity and resource efficiency [59,60,61]. Albrecht et al. (2017) [25] showed that rootstocks, derived from seedlings, develop a well-defined primary root, and have a greater proportion of root mass and the lowest shoot-to-root ratio compared to clonally propagated rootstocks. Increased root biomass could lead to an increase in the aboveground biomass of the plant. For our study, this means that cannabis plants derived from seedling rootstocks (SAR), or potentially vigorous rootstocks (PVR), should perform better in terms of measured morphological traits (fresh weight and bud compactness index) than cannabis plants derived from potentially dwarfing rootstocks (PDR). This assumption was not confirmed, as the average UIN for both scion genotypes grafted on PVR gave the lowest values (118 for FW and the same BCI). However, our result is in line with the observation of Purdy et al. (2022) [35], who found that the size of the root system does not consistently correlate with cannabis yield. As for FW, the highest average UIN for both scion genotypes reached SAR (127), confirming the hypothesis of the dominance of the root system with a well-developed primary root.

Some authors suggest that the restricted passage through the grafting union, in specific grafting combinations, is directly related to anatomical changes in the tissue at the grafting union [62], which are thought to be partly due to the restriction of polar transport of auxin (IAA). Grafting prevents the passage of IAA into the root system, which could stimulate the synthesis of cytokinins and consequently increase the vigor of the scion [63]. That could also explain increased vigour using PDR in our case. On the other hand, it is assumed that a reduced IAA concentration in the root system reduces the ratio between xylem and phloem in some plants, which may result in dwarfing of the scion [64,65]. Reduced rootstock growth may also be related to the accumulation of IAA in the rootstock and reduced cytokinin synthesis. An excessively high concentration of IAA causes oxidative stress and triggers degenerative processes in the root system [66]. Rootstocks also differ in the concentrations of other hormones, such as ABA and its derivatives—gibberellins GA9, GA19, and GA53 [67]. Hormonal profiles often show no direct correlation with growth parameters. Certain distant scion signaling proteins such as SlCyp1 can influence root system and vascular development. All this suggests a complex signaling between different rootstocks and scions that needs to be further investigated in cannabis plants [68].

In our study, the BCI was statistically significant only for the scion ‘ScionII’, with all three genotypes of the PDR type having the highest BCI values (0.25–0.29 g/cm) and the control combination ‘Control/ScionII’ the lowest (0.12 g/cm). In contrast, the PDR type had the greatest negative effect on BCI for the ‘ScionIII’ scion genotype, demonstrating the variability in the effects of different rootstock types on the scion genotype. Increased BCI could be one of the desired morphological improvements of cannabis plants through grafting to increase yields in indoor cannabis cultivation.

Manipulation of the defense mechanism of cannabis plants is crucial as it can lead to changes in the content of secondary metabolites [69]. Grafting has been shown to influence biosynthetic pathways and their accumulation [39,70]. There are several transport and signaling mechanisms between the rootstock and the scion that promote shoot adaptation to stress factors through the transport of signaling molecules involved in the plant defence mechanism. Long-distance signaling molecules may be responsible for the induction of systemic resistance in grafted plants [71]. The biosynthetic pathways of cannabinoids and terpenoids share a common precursor, isopentenyl pyrophosphate (IPP). IPP is synthesized either via the mevalonic acid pathway in the cytosol (MVA) or the plastidial methylerythritol pathway (MEP) [72,73]. The MEP pathway is posited to play a crucial role in the synthesis of cannabinoid-terpenoid compounds [74]. IPP acts as a substrate for the synthesis of geranyl diphosphate for monoterpenes, farnesyl diphosphate for sesquiterpenes, and geranylgeranyl pyrophosphate (GGPP) for tetraterpenes. Notably, GGPP is the immediate precursor for carotenoids [75,76]. The selection of specific rootstocks has been demonstrated to significantly enhance both the qualitative and quantitative carotenoid profiles, evidenced by increased concentrations of β-carotene and lycopene [37,38,40]. Given the shared biosynthetic pathways between cannabinoids and terpenoids, it can be hypothesized that grafting may stimulate the production of desired secondary metabolites in cannabis. Our data showed an effect of rootstock on the biochemical composition of cannabis plants, especially on the content of tCBD and tTHC. The effect was more pronounced in the ‘ScionIII’ genotype, which belongs to chemotype III and has a genetic predisposition for a high CBD content. Potentially dwarfing rootstocks (PDR) caused the highest increase in tCBD content (27%), from 10.1% (non-grafted) to 12.8% (average content for ‘ScionIII’ on PDR). However, as the ratio of CBD to THC is fixed in cannabis [77], the tTHC content also increased at almost the same value (29%), so the ratio CBD:THC was not changed.

Terpenes are the second most important group of biochemicals in cannabis and their expression varies greatly depending on genotype and growing conditions [1]. We observed differences in terpene content between different rootstock types (Tables S2 and S3). However, the terpene content was low overall. To investigate the influence of rootstock type on terpene content in more detail, different genotypes (e.g., of medical cannabis) should be included in further studies.

The CBD yield (CBDY) is determined by the CBD content and the yield of the inflorescences (IY). Since cannabinoids are genetically determined and the time window for their manipulation is relatively narrow, CBDY is mainly influenced by IY. This study has shown that IY can be increased in grafted cannabis plants by the grafting technique. In the genotype ‘ScionIII’, which naturally grows faster and bushier, grafting led to an increase in IY of 20–22% and in the genotype ‘ScionII’, which grows more upwards and has fewer side shoots, up to 71% higher IY was observed. This means that less vigorous scions can significantly increase their IY potential when grafted onto a suitable rootstock.

An increase in IY led to an increase in CBDY in this study, with the highest CBDY per plant causing PDR (average 4.8 g CBD/plant) for the genotype ‘ScionIII’. This was 55% higher than the non-grafted control plants. This is not in line with Purdy et al. (2022) [35], who found no increase in CBDY in grafted plants with a yield of up to 2.4 g CBD/plant. On the contrary, they even found a decrease in CBDY in one graft, which they explained because of the lower biomass yield of this graft combination. For the ‘ScionII’ genotype, the relative comparison showed an even greater increase in CBDY (up to 83% for SAR). However, absolute values of CBDY per plant were much lower due to the characteristics of the ‘ScionII’ genotype (chemotype II), which belongs to industrial hemp. Given the prevailing market prices for CBD isolate ($952/kg–$1964/kg) [78], graft-induced CBDY enhancement could drastically improve the efficiency of cannabidiol production.

Although the final yield of CBD depends on many factors during plant growth, determining the efficiency of CBD production begins with the efficiency of obtaining plants for flowering. When preparing grafted cannabis plants for CBD production, the critical step in evaluating the success of this new two-step grafting propagation method. This means how many rootstocks are needed at the beginning of the propagation process to obtain the desired number of plants for cultivation (CBD production). The number of rootstocks prepared is very important as these rootstocks require space and care, which incurs costs for electricity, growing space, and labour. Indoor design metrics such as electrical power, PPFD, efficiency, and photon conversion efficiency (g/mol) are the main drivers of electricity costs associated with CBD-rich biomass production and should be spent wisely [79]. Production Efficiency of cannabidiol index (PE_CBD_) was established in this study to evaluate the efficiency of CBD production using different types of rootstocks. The PE_CBD_ index covers the entire production process, from rootstock preparation to CBD yield. The survival rate of the rootstock has the greatest influence on the calculation and follows the paradigm: the higher the survival rate of the rootstock, the higher the CBD yield at the end of the cultivation process.

The PE_CBD_ revealed that the seedlings in this study stood out as the most efficient rootstock for cannabis production with average UIN values of 252 and 216 for ‘ScionII’ and ‘ScionIII’, respectively. The reasons for these high values are: (i) the high germination rate of the seeds ensured 100% establishment of the rootstock to be used for grafting and (ii) the higher survival rate of SAR grafts (40–70%, Table 1 and Table 2), which means that more plants are available for CBD production, resulting in higher total CBD yields of a SAR rootstock type, notably affecting the PE_CBD_ value. ‘ScionIII’ proved to be more receptive to grafting compared to ‘ScionII’. The considerable indoor space required for large-scale production is a challenge, especially when traditional cloning takes up 20–25% of the production area [80]. Using the one-step tailor-made grafting methodology [35], this demand could increase greatly as both scions and rootstocks need to be vegetatively propagated. In this respect, the use of SAR seems even more promising, as it reduces costs (eliminating the maintenance of mother plants and the preparation of clones) and increases PE_CBD_ (a higher survival rate of SAR plants is expected). Although our results have shown that SAR has a high potential for practical application, some disadvantages (e.g., high variability among seedlings, risk of formation of unwanted rootstock shoots at flowering time) should also be considered and further researched.

## 4. Materials and Methods

### 4.1. Cannabis Varieties

The grafting trial comprised five cannabis strains: the Slovenian population ‘Simba’ (with genotypes ‘S26’, ‘S63’, ‘S39’ and seedlings), the dioecious Hungarian variety ‘Tiborszallasi’ (genotype ‘T34’ and seedlings), the dioecious Italian variety ‘Carmagnola’ (genotypes ‘C23’ and ‘C45’), the monoecious Slovenian variety ‘Fiona’ (seedlings), and the feminized CBD variety ‘Merlot’ (genotype ‘MER’).

### 4.2. Rootstock Types and Scion Genotypes

Three rootstock types were used in this study, namely potentially dwarfing rootstocks (PDR), potentially vigorous rootstocks (PVR), and seedlings-as-rootstocks (SAR). The first two types were derived as clones from mother plants selected based on morphological and biochemical characteristics determined in our previous independent field trials. Prior to the present grafting research, the genotypes had not been utilized as rootstocks; thus, their effects on the scion were merely hypothesized. The third rootstock type (seedling) was obtained from seeds of selected cultivars. A comprehensive overview of the genotypes used for the rootstocks can be found in Table 4. The genotype ‘S39’ of the population ‘Simba’ and the genotype ‘MER’ of the cultivar ‘Merlot’ were used for the scions. Our previous field trials have shown that the ‘S39’ genotype belongs to chemotype II with a CBD and THC content of ≈2.7% and ≈2.5%, respectively, which is why it is called ‘ScionII’. The genotype ‘MER’ belongs to chemotype III with a ratio of THC (≈0.5%) to CBD (≈15.0%) <1, hence it is called ‘ScionIII’.

Combinations—each of the two scions (‘ScionII’ and ‘ScionIII’) were grafted onto the respective type and genotype of the rootstock. This resulted in six unique combinations for each rootstock type (Table 5). As a control, non-grafted scions were also grown as clones and used in this experiment. For each combination of scion and rootstock, up to 13 replicates of a particular rootstock genotype were prepared. A detailed description of the replicates can be found in the Results chapter.

### 4.3. Two-Step Grafting Method

**Figure 10:**
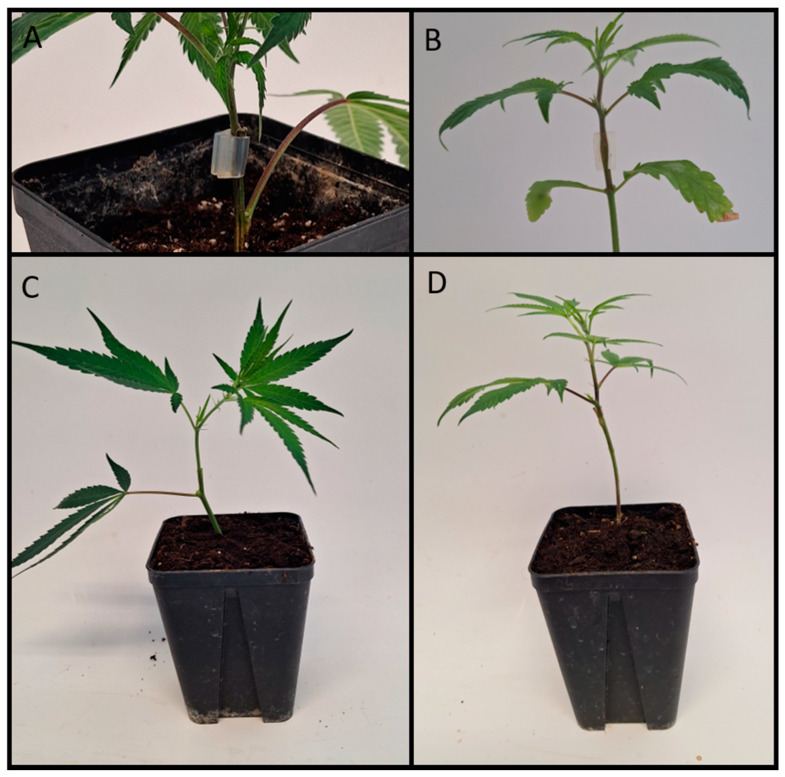
Grafted cannabis plants. (**A**) Scion grafted onto a clonally propagated rootstock after transplantation into 0.5 L pots. One remaining leaf on rootstock and grafting union can be observed. (**B**) Scion grafted onto seedling used as rootstock after transplantation into 0.5 L pots. Two remaining leaves from first node and grafting union can be seen. (**C**,**D**) Grafted plants with clonally propagated rootstock (**C**) and a seedling as rootstock (**D**) one week after transplantation—leaves of first node have fallen off.

Vegetatively propagated cuttings were taken from lateral shoots of the mother plants. A 13–16 cm shoot was cut at a 45-degree angle using sterilized scissors. This cut segment was immersed in Clonex hormone gel (Growth Technology, https://www.growthtechnology.com/, accessed on 5 January 2024), and then positioned upright in rockwool cubes (Rockwool Starter Cubes 1 1/2″ Large, https://cultilene.com/, accessed on 5 January 2024) for vegetative growth. Lateral leaves were removed, retaining only two–three leaves at the shoot’s apex. The dome was regulated to a stable temperature of 25 °C, a relative humidity between 95% and 100%, light intensity at 120 µmol m^−2^ s^−1^, and a photoperiod involving 18 h of light and 6 h of darkness (18L:6D). The density of the cuttings was established at 462 per m^2^. After a week, the relative humidity was decreased to 85% to 90%, while the other conditions remained unchanged. During the third week of rooting, relative humidity was further reduced to 75% to 80%. Depending on the availability of shoots, twelve to thirteen clones per rootstock genotype were created. Clones, fully rooted and approximately 21 days old, were used as rootstocks for grafting. All leaves of the rootstock were removed except the lowest one. The stem of the rootstock was cut approximately 2 cm above the lowest leaf. A sterilized scalpel made a vertical cut from the stem’s top to the point where the scion would be affixed. The scion, freshly cut from the mother plant (either ‘ScionII’ or ‘ScionIII’), was around 5 cm in length and had all but the top-most cluster of leaves removed. The scion’s bottom tip was cut into a matching wedge shape to fit into the approximately 1 cm incision in the rootstock. The graft was stabilized using a silicone grafting clip. The grafted clones were placed in propagation domes and sprayed with water daily for four days to maintain relative humidity around 100%. Starting from the fifth day, the dome’s humidity was gradually decreased. The fusion of the grafts was visibly noticeable after six days. Successful grafted clones were transplanted to 0.5 L pots 10 days post-grafting (Figure 10A). Regarding the seedlings used as rootstocks, seeds from three different varieties were sowed in germination trays and nurtured under glasshouse conditions. After three weeks of vegetative growth, the plants were cut approximately 2 cm above the first node without removing any leaves (Figure 10B). The rest of the grafting procedure followed the previously described method.

### 4.4. Vegetative and Flowering Growing Conditions

The mother plants were cultivated in the glasshouse of the Department of Agronomy at the University of Ljubljana (Ljubljana, Slovenia). A combination of natural light and supplemental high-pressure sodium (HPS) lighting was used to maintain an 18 h light and 6 h dark cycle (18L:6D) to promote vegetative growth. The greenhouse environment was controlled to a day/night temperature of 26 °C/18 °C and humidity between 50% and 70%. Aptus nutrients (https://aptus-holland.com/, accessed on 5 January 2024) were used to irrigate the plants, dosed according to the manufacturer’s recommendations.

The grafted plants were first potted in 0.5 L pots filled with substrate (Klasmann Substrate 5, https://klasmann-deilmann.com/en/, accessed on 5 January 2024). After 2 weeks of vegetative growth, the plants were transplanted into 10 L containers filled with a 30:70% mixture of perlite and substrate. The vegetative growth phase was carried out in a completely randomized design on a single bench under glasshouse conditions. For the flowering phase, the plants were relocated to a specialized cannabis flowering room. This room was equipped with artificial lighting (4× Sylvania SHP-TS 600 W HPS, 4× Sylvania SHP-TS 400 W HPS) that set up a 12-h light-dark cycle. The room’s environmental conditions in the room were set to a day/night temperature of 30 °C/25 °C and a humidity of 35% to 50%. The flowering experiment was conducted on two benches in the flowering room in a randomized block design. During the entire flowering stage, the plants were watered weekly with the Aptus Nutrient Schedule—PRO.

### 4.5. Harvest Method 

At harvest, plants were cut at the base, with their fresh weight and height measurements subsequently logged. Second-order branches were sampled for cannabinoid and terpene analyses. Leaves and stems of the inflorescences were detached, and the inflorescence sample was instantly preserved in liquid nitrogen and stored at −85 °C until ready for the lyophilization process. The samples were weighed post-lyophilization. The inflorescence density was evaluated using a slightly modified method inspired by Kakabouki et al. (2021) [81]. For each replicate, main inflorescences exactly 15 cm in length were weighed without leaf removal. Each inflorescence was then detached from the stem, hand-trimmed, and weighed once more. The trimmed inflorescences were dried at a steady temperature of 40 °C until no additional weight reduction was noted (roughly after two days), then reweighed. The remainder of the plant was dried separately under identical conditions. Post-drying, the plants were hand-trimmed, and the inflorescences were excised from the stems and weighed, along with the stems. All pertinent data were documented.

### 4.6. Analysis of Secondary Metabolites

#### 4.6.1. Analysis of Cannabinoids

For the identification and quantification of cannabinoids, we used a modified method of Adams et al. 2007 [82]. Samples of inflorescences were taken 8 and 12 weeks after flower induction for the scions ‘ScionII’ and ‘ScionIII’, respectively. Supporting stems and leaves were removed and the remaining inflorescences were homogenized to a fine powder. Cannabinoids were extracted from 500 mg subsamples with a mixture of chloroform (CHCl_3_) and methanol (MeOH) at a ratio of 9:1 (*v*/*v*) using an ultrasonic water bath for 15 min. The extracts were then filtered through 0.45 μm filters. The filtered extracts were diluted in a mobile phase with acetonitrile (C_2_H_3_N) and 0.1% acetic acid (CH_3_COOH) (*v*/*v*). The extracts were analyzed using an Agilent Technologies (Agilent Technologies Inc., Santa Clara, CA, USA) 1100 series HPLC chromatograph equipped with a Supelco Ascentis^®^ Express (Sigma-Aldrich, St. Louis, MO, USA) 5 μm C18 column. The injection volume was 20 μL and the flow rate was 1.0 mL/min. The oven temperature was set to 25.0 °C and the wavelength of the detection signal (λ) was 220 nm. The mobile phase was used as described above. Cannabinoid content was determined by measuring 11 cannabinoids, including CBDA, CBD, CBGA, CBG, cannabidivarinic acid (CBDVA), cannabidivarin (CBDV), THCA, Δ9-THC, Δ8-THC, tetrahidrocannabivarin (THCV), and cannabinol (CBN), using specific standards for each cannabinoid. Only the two most frequently occurring (tCBD and tTHC) were selected for presentation. The conversion factor 0.877 for CBDA and THCA was used when the carboxylic acid derivatives (CBDA and THCA) were added with the non-carboxylic acid forms (CBD and THC) to the concentrations of total cannabinoids (tCBD and tTHC).

#### 4.6.2. Terpene Analysis

For terpene analysis, terpenes were extracted from 200 mg subsamples with *n*-hexane in an ultrasonic water bath for 15 min. The extracts were then centrifuged and filtered through 0.45 μm filters. Terpene analysis was performed using a 6890 gas chromatograph (GC; Agilent Technologies Inc., Santa Clara, CA, USA) with a 973 network mass detector (MS; Agilent Technologies Inc., Santa Clara, CA, USA). The analysis was performed according to a method described by Gallily et al. (2018) [83] with modifications using a Rxi-35 Sil MS capillary column. Helium was used as the carrier gas at a constant flow rate of 1.2 mL/min. The temperature program started at 50 °C (held for 2 min) and then increased to 250 °C at a rate of 9 °C/min. The post-run temperature was set to 280 °C for 3 min. The mass spectrometer was set to the automatic scan mode of 50–550 amu. The ionization voltage was set to 1200 V, and the ion source and quadrupole temperatures were set to 230 °C and 150 °C, respectively. The terpenoid content was determined by measuring 19 terpenoids, including α-pinene, camphene, β-pinene, β-myrcene, 3-carene, α-terpinene, D-limonene, O-cymene, β-ocimene, γ-terpinene, terpinolene, linalool, isopulegol, geraniol, β-caryophyllene, α-humulene, nerolidol, guaiol, and α-bisabolol, using specific standards.

High-performance liquid chromatography (HPLC) and gas chromatography–mass spectrometry (GC-MS) analyses were performed at the Faculty of Health Sciences, University of Ljubljana (Ljubljana, Slovenia). Cannabinoids and terpenes were quantified using the standard calibration curve. Terpenes were identified using the library (HPCH2205, Wiley7N in FENSC3) [82].

### 4.7. Statistical Analysis

Nine different rootstock genotypes and two scion genotypes were used in this study. Non-grafted scions were also used. Due to the large differences in scion characteristics, scions were not compared with each other, and rootstock genotype was used as the only factor within each scion genotype. Six variables were subjected to one-way ANOVA analysis, namely plant fresh weight, bud compact index, CBD and THC content, inflorescence yield and CBD yield. Prior to analysis, each response variable was tested for the assumptions of normal distribution and homogeneity of combination variances using Levene’s test. Significant differences in means indicated by ANOVA were assessed using Duncan’s test (α = 0.05). All statistical analyses were performed using the agricolae package in the statistical software program R version 3.2.5 [84]. The graphs were created using Microcal software Version 6, Origin 6.0 Working Model, 1999 [85].

### 4.8. Efficiency of CBD Production

The production efficiency of cannabidiol (PE_CBD_) was determined based on the number of prepared rootstocks. The total cannabidiol yield of a given rootstock genotype (tCBDY) was calculated by multiplying the number of surviving grafted plants (SGP) by the average cannabidiol yield per plant (CBDY). The total cannabidiol yield per rootstock (YRCBD) was calculated by dividing tCBDY by the number of rootstocks prepared for each combination (NR). The efficiency quotient of CBD production (PE_CBD_) was calculated using the formula below (Formulas (1)–(3)) and expressed as an Unweighted Index Number (UIN).

Formulas (1)–(3): The efficiency quotient of CBD production (PE_CBD_) calculation. Formula (1): tCBDY—total cannabidiol yield of scion grafted on some rootstock genotype, SGP—survived grafted plants, CBDY—average cannabidiol yield per plant. Formula (2): YR_CBD_—total cannabidiol yield per rootstock, NR—number of rootstocks prepared for each combination. Formula (3): PE_CBD_—efficiency quotient of CBD production.

(1) tCBDY=SGP×CBDY

(2) $${\mathrm{YR}}_{\mathrm{CBD}}\;=\frac{tCBDY}{NR}$$

(3) $${\mathrm{PE}}_{\mathrm{CBD}}\;=\frac{{YR}_{CBD}\;treatment}{{YR}_{CBD}\;control}\times 100$$

## 5. Conclusions

This study showed that rootstock type influences the survival rate, morphological parameters, and biochemical composition of cannabis plants. A relative comparison between grafted and non-grafted plants showed better characteristics of the grafts in most cases, indicating the great potential of the grafting technique for cannabis production. Optimizing rootstock selection and grafting techniques, understanding the mechanisms behind the observed morphological changes, and a deeper exploration of the influence of rootstock on the biochemical composition and yield of cannabis plants are some of the topics for future research. Grafting is a new cultivation method in cannabis production and requires additional equipment, space, time and “experienced hands”. As the cannabis industry continues to expand, research in these areas will be of increasing importance in maximizing the potential of this plant. Further research on cannabis grafting is necessary, focusing on the physiological processes in the scion, changes in hormone balance, metabolic pathways, and genetic analyses, such as changes in gene expression in specific rootstock-scion combinations. In addition, priority should be given to breeding rootstocks that are resistant to biotic and abiotic factors to improve the success of grafting and the resistance of the plants.

## Figures and Tables

**Table 1:** Rootstock rooting success rate and survival of grafted and non-grafted plants for ‘ScionII’ scion genotype.

Plant Material/ Combinations	Number of Rootstocks (NR)	Number of Successfully Rooted Rootstocks (RR)	Number of Survived Plants (SP)
’Vigor-C23/ScionII’	13	7	5
’Vigor-S26/ScionII’	13	12	3
’Vigor-S39/ScionII’	13	9	5
’Dwarf-C45/ScionII’	13	6	2
’Dwarf-S63/ScionII’	13	6	4
’Dwarf-T34/ScionII’	13	6	3
’Seedl-TIB/ScionII’	10	10	4
’Seedl-FIO/ScionII’	10	10	7
’Seedl-SIM/ScionII’	10	10	6
’Control/ScionII’	12	5	5

SIM, S—population ‘Simba’, FIO—variety ‘Fiona’, TIB, T—variety ‘Tiborszallasi’, C—variety ‘Carmagnola’, Seedl—seedling rootstock type, Vigor—vigorous rootstock type, Dwarf—dwarfing rootstock type.

**Table 2:** Rootstock rooting success rate and survival of grafted and non-grafted plants for ‘ScionIII’ scion genotype.

Plant Material/ Combinations	Number of Rootstocks (NR)	Successfully Rooted Rootstocks (RR)	Survived Plants (SP)
’Vigor-C23/ScionIII’	12	5	3
’Vigor-S26/ScionIII’	12	11	3
’Vigor-S39/ScionIII’	13	8	2
’Dwarf-C45/ScionIII’	12	6	3
’Dwarf-S63/ScionIII’	12	6	3
’Dwarf-T34/ScionIII’	12	10	3
’Seedl-TIB/ScionIII’	10	10	4
’Seedl-FIO/ScionIII’	10	10	4
’Seedl-SIM/ScionIII’	10	10	5
’Control/ScionIII’	12	3	3

SIM, S—population ‘Simba’, FIO—variety ‘Fiona’, TIB, T—variety ‘Tiborszallasi’, C—variety ‘Carmagnola’, Seedl—seedling rootstock type, Vigor—vigorous rootstock type, Dwarf—dwarfing rootstock type.

**Table 3:** Average bud compact index with corresponding standard deviations (SD) of grafted and non-grafted plants for ‘ScionII’ scion genotype.

Plant Material/Combination	BCI (g/cm ± SD)	*p* ≤ 0.05 ^1^
‘Dwarf-C45/ScionII’	0.2960 ± 0.012	a
‘Dwarf-T34/ScionII’	0.2669 ± 0.042	ab
‘Dwarf-S63/ScionII’	0.2445 ± 0.053	ab
‘Vigor-S26/ScionII’	0.2209 ± 0.026	ab
‘Seedl-SIM/ScionII’	0.2092 ± 0.064	abc
‘Seedl-TIB/ScionII’	0.1980 ± 0.088	bc
‘Vigor-C23/ScionII	0.1810 ± 0.070	bc
‘Vigor-S39/ScionII’	0.1792 ± 0.069	bc
‘Seedl-FIO/ScionII’	0.1766 ± 0.032	bc
‘Control/ScionII’	0.1225 ± 0.042	c

^1^ Different letters indicate statistically significant difference between varieties or combinations (Duncan, *p* ≤ 0.05).

**Table 4:** Morphological characteristics of mother plants clones for dwarfing and vigorous type of rootstock and seedling rootstocks derived from seeds of listed varieties.

Rootstock Type	Variety—Genotype	Height	Stem Thickness	Natural Branching
Vigorous	Carmagnola—C23	High	High	Low
	Simba—S26	High	High	High
	Simba—S39 *	Mid	High	Low
	Merlot—MER *	Mid	High	High
Dwarfing	Carmagnola—C45	Low	Low	Low
	Simba—S63	Low	Low	High
	Tiborszallasi—T34	Low	Low	High
Seedlings	‘Tiborszallasi’ seedlings	Specific characteristics of the variety		
	‘Fiona’ seedlings			
	‘Simba’ seedlings			

* Used as scion.

**Table 5:** Combinations of rootstocks and scions carried out in experiments with entitled marks.

Potentially Vigorous Rootstock (PVR)			Potentially Dwarfing Rootstock (PDR)			Seedling-as-Rootstock (SAR)		
Rootstock Genotype	Scion Genotype	Combination Name	Rootstock Genotype	Scion Genotype	Combination Name	Rootstock Genotype	Scion Genotype	Combination Name
C23	ScionII	Vigor-C23/ScionII	C45	ScionII	Dwarf-C45/ScionII	Tiborszallasi seedlings	ScionII	Seedl-TIB/ScionII
S26		Vigor-S26/ScionII	S63		Dwarf-S63/ScionII	Fiona seedlings		Seedl-FIO/ScionII
S39		Vigor-S39/ScionII	T34		Dwarf-T34/ScionII	Simba seedlings		Seedl-SIM/ScionII
C23	ScionIII	Vigor-C23/ScionIII	C45	ScionIII	Dwarf-C45/ScionIII	Tiborszallasi seedlings	ScionIII	Seedl-TIB/ScionIII
S26		Vigor-S26/ScionIII	S63		Dwarf-S63/ScionIII	Fiona seedlings		Seedl-FIO/ScionIII
MER		Vigor-MER/ScionIII	T34		Dwarf-T34/ScionIII	Simba seedlings		Seedl-SIM/ScionIII
Control combinations								
Control/ScionII: non-grafted clones of ScionII scion genotype								
Control/ScionIII: non-grafted clones of ScionIII scion genotype								

SIM, S—population ‘Simba’, FIO—variety ‘Fiona’, TIB, T—variety ‘Tiborszallasi’, C—variety ‘Carmagnola’, Seedl, SAR—seedling-as-rootstock combination, Vigor, PVR—potentially vigorous rootstocks, Dwarf, PDR—potentially dwarfing rootstocks, ScionII—scion of chemotype II, and ScionIII—scion of chemotype III.

## Data Availability

All data from this study are included in this published article and as Supplementary Materials.

## Supplementary Materials

The following supporting information can be downloaded at: https://www.mdpi.com/article/10.3390/plants13081117/s1, Table S1: Results of the real measurement of the experiment with three types of rootstocks and two genotypes of scions; Table S2: Terpene content in combinations of rootstocks with the ‘ScionII’ genotype; Table S3: Terpene content in combinations of rootstocks with the ‘ScionIII’ genotype.
